# Preventing Dog Bites: It Is Not Only about the Dog

**DOI:** 10.3390/ani10040666

**Published:** 2020-04-11

**Authors:** Laura A. Reese, Joshua J. Vertalka

**Affiliations:** 1 Urban and Regional Planning and Political Science, Michigan State University, Human Ecology Building 552 W. Circle Drive, Room 208A, East Lansing, MI 48824, USA; 2 Global Urban Studies, Michigan State University, 509 E. Circle Drive, RM 447, East Lansing, MI 48824, USA; joshua.j.vertalka@gmail.com

**Keywords:** dog bites, dog bite prevention, dog breeds and bites, public health, victim behavior

## Abstract

**Simple Summary:**

Dog bites can have an array of negative health impacts on human victims. Research on the correlates of bites focused on limited sets of variables and produced conflicting findings. Data were drawn from police department reports of dog bites in the city of Detroit between 2007–2015. This project expands knowledge about the correlates of dog bites by exploring a comprehensive set of variables related to the breed type of dog, the nature of its surroundings, and the circumstances surrounding the bite. The greatest risk of bites does not come from wandering feral dogs, nor is it primarily related to the nature of the victim. Based on multiple regression, the victim was most likely bitten in their own yard by a single neighborhood dog that escaped from its home or yard. Human error often contributes to bites.

**Abstract:**

Background: Dog bites can have an array of negative health impacts on victims. Research focusing on the correlates of bites focused on limited sets of variables and produced conflicting findings. Objective: To expand knowledge about the correlates of dog bites by exploring a comprehensive set of variables related to the nature of the dog and the circumstances surrounding the bite not commonly explored in extant research. Methods: Data were drawn from police department reports of dog bites in the city of Detroit between 2007–2015; 478 dog bites were reported. Multiple regression was used to determine the significant correlates of dog bites, focusing on the nature of the dog and the circumstances surrounding the bite. Results: Bites were caused by a neighborhood dog. Thirty-two percent of the reports involved dogs running loose; 25% dogs that had escaped from a fenced or unfenced yard; 9% escaped from their home; and 8% had broken off a chain, were being walked, or were in their own home. Based on multiple regression, the victim was most likely bitten in their own yard by a single neighborhood dog that escaped from its home or yard. Breed of dog was not correlated with bites in multiple regression. Conclusions: The greatest risk of bites does not come from wandering feral dogs. Based on multiple regression, the victim was most likely bitten in their own yard by a single neighborhood dog that escaped from its home or yard. Human error often contributes to bites.

## 1. Introduction

Health impacts of dog bites include: rabies; infection; disfigurement, particularly for children as they are more likely injured in the head and face; mobility issues; amputation; full or partial paralysis; death; fear of animals; fear of walking to school or in the neighborhood; reduced outdoor exercise; post-traumatic stress; and other psychological or emotional issues (depression, withdrawal, sleep disturbances, flashbacks, and phobias about dogs or other animals) [1,2,3].

An increasing body of literature, across several disciplines, has examined the correlates of and the potential preventative measures for dog bites in humans. While informative, the research tended to focus on limited sets of variables, such as the nature of the victim (mostly age and gender) and the nature of the dog involved (breed, size, sex, and sterilization status). This research contributes to extant knowledge about dog bites by using police department bite incident data to explore a wider set of potential explanatory variables, including the traits of the dog involved, but also the broader circumstances surrounding the bite. It employs data on 478 bites, over a period of eight years, in an urban setting that includes significant numbers of roaming dogs (both feral and owned), and incorporates a number of variables not included in past research. To be clear, this research focuses on dog bites as an expression of aggressive behavior that may result from fear, human interactions, health issues, or other situational factors. It does not include natural dog behavior, such as bites that occur when playing or other accidental contact. 

An extensive focus of research has been on which dogs are most likely to bite, a large amount of it related to “breed.” Efforts to link breed with bite propensity are problematic for a number of reasons: all dogs can and do bite and the environment or other personality traits of the dog appear to be better predictors of biting than breed [4,5]; bite propensity by breed is extremely difficult to calculate because valid measurement requires that the population of particular breeds in the area of study be known [6]—observers, victims, and public safety officials are notoriously poor at estimating dog breeds. This is also true, to a lesser extent, among those in animal-related professions, especially regarding shelter dogs, where it is common to use visual appearance to recognize breed instead of genetic testing that could be economically unaffordable [7]. Researchers found that German Shepherds and cross-breed dogs accounted for the majority of bites over time; dogs on the “dangerous dog list” including American Staffordshire and Pit Bull Terriers were the perpetrators of bites in only a small number of cases [8]. Other scholars found similar results, in that the types of dogs banned in various countries and municipalities did not appear to cause the largest proportion of bites [9]. 

Other research pointed to unneutered male dogs as the most frequent perpetrators of bites [10,11], with castration reducing the risk [12]. Other dog traits linked to bites included the size of the dog, with larger dogs more likely to bite and with greater severity [13]. Dogs that experienced aversive training methods also appeared more like to exhibit aggression toward strangers [14]. 

However, research found conflicting results on these issues. For example, smaller dogs and those that have been acquired for companionship, rather than protection, were found to be more likely to bite [11]. Additionally, dogs that have been spayed or neutered may actually show increased fear and aggression, particularly if castration was performed at a younger age [15,16]; aggression in free-roaming male dogs may not change after castration [17]. 

In most cases, however, it is the interaction between human behavior and dog traits that creates risk, such as leaving the dog chained in the yard, other types of under- and improper socialization, and actions of the victim such as harassing or restraining the dog [11,18]. Human actions, particularly those of children, often lead to bites as they can be interpreted by dogs as threatening or are simply surprising or unexpected [19]. This might include sticking hands through a fence or into a kennel; taking away a toy, food, or some other “resource”; teasing a chained or tethered dog; or, trying to pet an unknown dog [20]. Thus, it appears that victims are more likely to initiate the contact that led to a bite than the dog [21]. Finally, some research suggests that most bites come from dogs owned by the victim [22,23]; other work found bites from roaming or unknown dogs most common [24]. To address inconsistencies in these findings this research addresses the following questions:

- Are bites more likely to come from own dogs, unknown roaming dogs, or neighborhood dogs not owned by the victim?
- In the context of feral roaming dogs, do bites appear be caused by groups of such dogs?
- Does presumed or estimated breed of dog appear to be related to bite incidents?
- Are there common sets of circumstances surrounding bites, such as tethering/chaining dogs in yards, harassing a dog, or other unintentional victim behavior that can be interpreted by dogs as threatening?

## 2. Materials and Methods

### 2.1. The Case

This research focused on the city of Detroit, where residents experience dog bites at rates higher than many other cities [25]. Compared to the 2008 Agency for Healthcare Research and Quality national estimates, the bite-related ER visits in the Detroit area (for 2010) were almost four times the rate of urban areas nationwide. The city also has high numbers of stray and feral dogs. While research modeling the US dog population assumed that the number of feral or unowned dogs was “negligible” [26], estimates of stray and feral dogs in Detroit ranged from 3000 to 50,000 [27]. Detroit also experienced extreme fiscal distress, which challenged its ability to provide animal welfare/control services [28,29]. The roaming animal problem, in particular, was exacerbated by foreclosures, vacancies, and structural abandonment which left habitats for stray and feral animals to shelter in and for illegal activities, such as dog fighting, to be conducted [30].

### 2.2. The Data

Data were drawn from police department reports in the category of “animal crimes,” which include dog bites, animals at large, and animal cruelty. For the purposes of this study, only the dog bite data were examined for the period between 2007–2015. Over this time, there were 478 dog bites reported to the police department in Detroit. Since many bites, particularly those that are minor or might be the result of a criminal act such as dog fighting, are not reported, it should be assumed that this number underestimates bites for the time period. Police reports were read by two individuals to establish the coding scheme, coded by a single individual to assure consistency, and then coding was randomly checked by one of the authors. The address of the incident was collected to allow for placement of within one of the 300 census tracts in the city. 

### 2.3. Variables

The bite data measured the dependent variable: the number of dog bites per census tract. Independent variables, selected based on the forgoing discussion of research included: breed estimate, based either on victim report or the assessment of responding officers; whether the dog was roaming; the dog was owned and got loose from its yard or home; whether the bite occurred in the home; the victim had engaged in behavior that provoked the dog; whether the dog was chained regularly or at the time of the incident; the number of dogs involved in the incident; whether the dog belonged to the victim, lived in the neighborhood, or was unknown to the victim; if the victim was working (as a mail carrier or utility worker); and whether the victim was walking in the neighborhood.

## 3. Results

### 3.1. Summary Statistics

Table 1 presents the frequency data for the most common conditions surrounding dog bites in the city. Overwhelmingly, bites were caused by a neighborhood dog, with only a single dog (84%) and victim (90%) involved. This suggested that the greatest risk of bites did not come from wandering feral dogs as was implied in local media [31]. Thirty-two percent of the reports involved dogs running loose; 25% dogs that had either escaped from a fenced or unfenced yard; 9% escaped from their home; and 8% had broken off a chain in their yard, were being walked by their owners, or were in their own home. Overall, 88% of the bites occurred out of doors. Often, police reports provided more detail about what the dog was doing prior to the bite. Many of the dogs that escaped their yards, even when there was a fence, were chained to something in the yard. 

A plurality of bites occurs to victims walking in a neighborhood, at the park, and going to stores (32%) although in 25% of the cases, the victims were in their own yards when loose dogs bit them. While the US Postal Service lists Detroit as one of the most dangerous cities for workers in terms of dog bites, only 10% of bites occurred while the victim was working, including police and animal control officers. In 6% of the cases the bite occurred as the victim was entering or visiting the home where the dog resided or was interacting with the dog in some way (trying to pet it, feed it, playing with it, and in the case of children, hitting it). The reports were explicitly examined for indications that the victim had harassed the dog in some way; in most cases (54%) this was unknown but in 42% of the incidents, actions on the part of the victim were noted as causing the bite. In 13% of incidents victim behavior likely contributed to the bite, but did not involve harassment. Such actions most often included running from a dog, feeding an unknown dog, or trying to pet or pick up an unknown dog.

As noted previously, much of the research on dog bites focused on “breed.” First, in all cases, police reports contained some estimate of the type of dog, with 48 different breeds and breed mixes represented. The breed types listed in the table were those most often noted in reports. It appeared that Pit Bull mixes and other bully types were the most likely to be indicated in the police reports (73%), followed by a category including Rottweilers, German Shepherds, Akitas and Chows (21%), dogs not as commonly thought to be aggressive mutts, Dalmatians, Boxers, Labs, and Bouviers (4%), and finally, “small dogs” (3%). There is evidence to conclude that bully breeds are more likely to be involved in bites simply because they are the most numerous dogs in the city 

### 3.2. The Dog and Circumstances of the Bite

Table 2 presents the results of the characteristics of the dog and the circumstances surrounding the bite regressed on the number of bites in a census tract. Based on the significant correlations in multiple regression, the victim was most likely bitten in their own yard by a single neighborhood dog that escaped from its home or yard. However, this regression produced a number of variables with high variable inflation factors (VIF), specifically involving whether the dog was in its own home and whether the victim was walking or working in the neighborhood (variance inflation factors quantify the extent of multicollinearity in OLS regression by providing an index that measures how much the variance of an estimated regression coefficient is increased because of collinearity. Values of more than 4 or 5 are often regarded as being moderate to high, while values of 10 or more are regarded as very high although these tend to be rules of thumb). These variables were removed from the model presented in Table 3.

After addressing issues of multicollinearity, the results were the same. The victim was most likely to be bitten in their own yard by a single neighborhood dog that got off a chain in the yard or otherwise escaped its home or yard. In this case, all VIF were under 5. These variables accounted for 77% of the variation in the number of dog bites within a census tract. 

## 4. Discussion

Much has been written in the media about the high numbers of roaming and feral dogs in Detroit, including concerns about the possibility of attacks on humans. The data here suggest that these concerns may be unwarranted or, at the least, over-stated. Dog bites are most likely to come from owned neighborhood dogs that have escaped their tethers, homes, or yards as opposed to unknown dogs. Victims are also most likely to suffer a bite in their own yards or close to home, again supporting the notion that known neighborhood dogs are most likely to bite. Breed of dog does not appear to be related to bite propensity based on multiple regression although, as noted, breed estimates by police, victims, or bystanders are notoriously faulty. 

Several policy implications logically emanate from the findings here. First, it appears that dog bites are most likely to result from some type of human error: allowing dogs to roam around the neighborhood and improper containment in yards or homes. Dogs that are chained in yards or otherwise kept outside often lack proper socialization and can become bite risks if they escape the yard or tether [11,19]. Dogs need to be properly socialized, ideally kept inside the home, with time outside spent in a secure yard or being walked by their owners on a leash. 

Humane education is needed to foster better human caretaking of owned dogs. Children and their families need to be educated about the proper care of dogs, how to best interact with them, and how to behave when faced with an unknown or roaming dog. Education programs that begin in schools and involve parents were found to increase general knowledge about the needs of animals, at least in the short term, although they may not affect actual interactions with animals [32]. Dog training programs are also important to provide owners necessary knowledge about dog behavior and wellbeing, as well as the instruments necessary to effectively manage possible behavioral problems. Programs to teach nonviolent or non-aversive methods to train pets have also been recommended as a way to reduce cruelty resulting from lack of knowledge [33]. Future research might explore whether the owners of dogs that had bitten had any knowledge about dog training or expected behavior, as well as their ability to predict when aggressive behavior toward people might occur so as to avert potentially dangerous situations. Any connections between breed and the extent and nature of training the owner has undertaken with the dog might also be explored. 

From a city prospective, while it is unlawful to tether dogs in yards beyond a limited time, there are insufficient numbers of animal control officers to effectively enforce the ordinance. Increased efforts to catch roaming dogs and/or remove dogs from owners leaving them chained in yards result in more dogs coming to the animal control shelter, which runs over capacity in the best of conditions [34]. Devoting increased resources toward animal control functions—both sheltering and ordinance and public health enforcement—would assist in reducing the conditions that lead to bites. 

## 5. Conclusions

In conclusion, four research questions developed from gaps in the extant literature were posed in this paper. These are summarized below:

- Under what conditions are dog bites most likely to occur? Are bites more likely to come from own dogs, unknown roaming dogs, or neighborhood dogs not owned by the victim?
  Dog bites appear to be most likely to result from neighborhood dogs that have gotten loose or are otherwise running free.
- In the context of feral roaming dogs, do bites appear to be caused by groups of such dogs?
  The evidence does not point to feral dogs as the most likely perpetrators of bites. Further, the preponderance of bites come from a single dog; attacks from roaming packs do not appear to be a common risk.
- Does presumed or estimated breed of dog appear to be related to bite incidents?
  No. While the majority of dog bite reports indicate a bully bread, breed identification is very faulty and there is no correlation between breed and bites in multiple regression.
- Are there common sets of circumstances surrounding bites, such as tethering/chaining dogs in yards, harassing a dog, or other unintentional victim behavior that can be interpreted by dogs as threatening?
  Police reports indicate that 42% of the victims had harassed the dog or provoked the bite. Coupled with the fact that loose owned dogs were those most likely to bite, it appears that both victim and owner error largely contribute to bite risk.

This research had several limitations. First, it was conducted in a single city which, because of its economic distress, may not be representative of many cities across the US. Second, the use of police bite data necessarily limited the analysis to those bites that were reported. These were likely to be more serious and less likely to involve dogs owned by the victim; thus, the data may undercount actual bites. Individual police officers likely vary in the tightness of their definitions and description of bites and the consistency with which definitions are applied. The breed estimate variable may be problematic due to the inability of individuals to accurately judge breeds, particularly with mixed-breed dogs; even if the officer saw the dog, it is far from certain that they would apply the same breed definition as their fellow officers. In the literature, free-roaming dogs are defined as feral or living on the streets, while loose dogs are those that are owned but have escaped the home, yard, or leash. Officers may apply these definitions differently as well. Police reports were coded for information regarding whether the dog bite was provoked in some way by victim behavior. If officers do not see the incident (most likely), they must rely on witness/victim statements, which may be faulty; there may be variation in the extent that individual officers attempt to investigate provocation further. Finally, victims and witnesses could be inaccurate in their reporting of whether the dog lived in the neighborhood or was completely unknown.

## Figures and Tables

**Table 1:** Frequency data: most common circumstances of reported dog bites *.

Variable	%
Whose dog was involved:	
Own dog	03
Neighborhood dog	83
Unknown dog	14
What dog was doing:	
Wandering at large	32
Escaped yard	25
Escaped house	09
Broke off chain	08
Being walked	08
In own home	08
What victim was doing:	
Walking outside	44
In own yard	19
Working	10
In the dog’s home or yard	06
Interacting with dog	06
Victim Harassed Dog:	
Unknown	54
Yes	42
Reported breed of dog:	
Bully mix	73
Larger dogs often considered to be aggressive	21
Larger dogs not typically considered aggressive	04
Small dogs	03

* Percentages may not equal 100 due to rounding.

**Table 2:** Regression, dog bites by nature of dog and circumstances.

Titles	B	Standard Error	T Value	Probability
Whose dog involved	0.27	0.07	3.59	0.00 **
Dog wandering at large	0.00	0.07	0.07	0.94
Pit Bull type	0.04	0.06	0.67	0.50
Number of dogs	0.27	0.07	3.76	0.00 **
Dog got off chain	0.16	0.09	1.77	0.08
Dog in own house/yard	−0.01	0.09	−0.07	0.95
Dog escaped house/yard	0.20	0.06	3.22	0.00 **
Victim was walking	−0.02	0.09	−0.17	0.87
Victim was in own yard	0.19	0.06	2.93	0.00 **
Victim was working	−0.03	0.08	−0.38	0.70
Victim harassed dog	0.02	0.06	0.37	0.71
Constant	0.01	0.08	0.18	0.86
Adjusted R^2^	0.77			

** significant at the 0.01 level.

**Table 3:** Reduced regression, dog bites by nature of dog, and circumstances.

Titles	B	Standard Error	T Value	Probability
Whose dog involved	0.26	0.07	3.65	0.00 **
Dog wandering at large	−0.00	0.07	−0.05	0.96
Pit Bull type	0.04	0.06	0.60	0.55
Number of dogs	0.27	0.07	3.81	0.00 **
Dog got off chain	0.14	0.07	1.90	0.06
Dog escaped house/yard	0.20	0.06	3.22	0.00 **
Victim was in own yard	0.18	0.06	2.94	0.00 **
Victim harassed dog	0.02	0.06	0.32	0.75
Constant	0.01	0.08	0.16	0.87
Adjusted R^2^	0.77			

** significant at the 0.01 level.
